# Global Perspective of the Management of Patients with Differences of Sex Development in Low- and Middle-Income Countries: A Scoping Review

**DOI:** 10.1159/sxd/aerag004

**Published:** 2026-09-10

**Authors:** Agustini Utari, Marcelo Silberkasten, Salwa Musa, Samar Hassan, Rajni Sharma, Wichor Bramer, Yvonne G. van der Zwan, Stenvert L.S. Drop

**Affiliations:** a Department of Pediatrics, Faculty of Medicine, Diponegoro University, Semarang, Indonesia; b Department of Clinical Psychology, Hospital de Niños Ricardo Gutierrez Buenos Aires, Buenos Aires, Argentina; c Pediatric Endocrine Department, Gaafar Ibn Auf Pediatric Tertiary Hospital, Khartoum, Sudan; d Department of Paediatrics and Child Health, Faculty of Medicine, Al-Neelain University, Khartoum, Sudan; e Division of Pediatric Endocrinology, Department of Pediatrics, All India Institute of Medical Sciences, New Delhi, India; f Medical Library–Erasmus MC, Erasmus University Medical Centre Rotterdam, Rotterdam, The Netherlands; g Department of Paediatrics, Isala Hospital, Zwolle, The Netherlands; h Division of Endocrinology, Department of Pediatrics, Sophia Children’s Hospital, Erasmus MC, Rotterdam, The Netherlands

**Keywords:** Disorders/differences of sex development, Management of difference of sex development, Molecular genetics of difference of sex development, Low- and middle-income countries

## Abstract

**Background:**

We explored literature published between January 2010 and July 2024 on the diagnosis and management of patients with differences of sex development (DSDs) in low-middle- and upper-middle-income countries (LMIC/UMIC).

**Summary:**

Scientific databases were searched, and peer-reviewed articles were reviewed according to predefined inclusion criteria. Data were extracted, including the study focus, geographical location, target population, target disease(s), and outcomes. Overall, 557 of the 627 papers addressed various aspects of DSD management and 20 were classified as reviews. Retrospective clinical studies (*n* = 121) and molecular genetic studies (*n* = 150) were mostly single-center observational studies that described various DSD conditions or focused on specific diagnoses. Case reports (*n* = 264) describing the clinical and molecular genetic aspects of DSD were prominent in middle-income countries. International collaboration was mentioned in 15% of studies. In-depth screening was conducted using a generative AI-based data extraction tool. A predefined extraction prompt was applied ensuring consistency. The screening resulted in a pragmatic impact grading A–E. Moreover, the inclusion of various molecular genetic tests contributed to improved diagnostic accuracy and diagnostic yield and reduction of misclassification and diagnostic delay. These tests led to changes in hormonal and surgical management and contributed to the avoidance of unnecessary interventions. Genital surgery remained controversial with respect to its appropriateness, timing, and long-term outcomes, leading to clinical uncertainty in the decision-making process. A pragmatic algorithm to the diagnosis and management of DSD suitable for low-resource settings is presented.

**Key Messages:**

The geographical distribution and volume of studies on DSD management in LMIC/UMIC originated in East/South Asia, Middle East/North Africa, and South/Central America. The inclusion of various molecular genetic tests contributed to improved diagnostic accuracy, with positive effects on hormonal and surgical management. We present a pragmatic diagnostic algorithm for the management of DSD suitable for low-resource settings with a key role for a multidisciplinary approach. We advocate the development of context-specific management guidelines tailored to LMIC/UMIC, considering sociocultural environments and resource constraints. Furthermore, promoting international collaboration in education, training, and consultation will be highly beneficial for improving the care of individuals with DSD in RLS.

## Introduction

Differences of sex development (DSDs) are an umbrella term encompassing a heterogeneous group of conditions characterized by a wide range of genital anomalies, which may manifest at birth or later in life. These conditions result in atypical genital morphology, pubertal development, and reduced reproductive capacity [1]. Its etiology is highly diverse, stemming from various chromosomal, genetic, and hormonal defects. The incidence of DSD among individuals with a 46, XY karyotype is estimated to be 1 in 20,000 births. For individuals with a 46, XX karyotype, the global incidence is estimated at 1 in 14,000–15,000 live births, although this may vary by region owing to ethnic differences [2]. In the past decade, an increasing number of genetic causes – both familial and de novo – affecting gonadal development have been identified [3–5].

In resource-limited settings, DSD management is hindered by limited expertise and resources, often resulting in delayed or incomplete diagnostic workups and treatment. In many parts of the world, the challenges associated with DSD management are further complicated by cultural and religious barriers, societal taboos, and public misconceptions [6–12]. Given the marked etiological heterogeneity and small number of patients per center or region, most studies are observational or descriptive in nature. Scoping reviews are particularly useful when the literature is complex and heterogeneous because they systematically map existing research, identify gaps in the literature, and define areas for future investigation [13]. This scoping review aims to evaluate the literature on the management of DSD published over a 13.5-year period (January 2010 to July 2024) originating from or pertaining to low- and lower-middle-income countries (LMICs) and upper-middle-income countries (UMICs). Specifically, the objectives were to identify reports of DSD research by geographic region, assess the type of evidence available, and examine how research on DSD management is conducted. Additional questions include whether regional differences exist in clinical and molecular genetic characteristics as well as in the challenges, trends, and outcomes related to management.

## Methods/Manuscript Selection Procedure

A literature search was conducted in July 2024 using various bibliographic databases (Embase.com, MEDLINE All via Ovid, Web of Science Core Collection, WHO Global Health Library, SciELO, CINAHL via EBSCOhost, and ERIC via Ovid) (see online suppl. Table 1; for all online suppl. material, see https://doi.org/10.1159/sxd/aerag004). However, the review protocol did not precede this search. The search was carried out by an expert librarian with a PhD and expertise in developing searches for systematic reviews [14, 15]. The search string included variations and synonyms of the following terms: disorders/differences in sex development (DSD), as well as specific disorders and anomalies classified under the umbrella term DSD, in association with the terms LMICs and UMICs.

For the year 2023, low-income economies were defined as those with a Gross National Income (GNI) per capita of USD 1,085 or less, as calculated using the World Bank Atlas method in 2021. Lower-middle-income economies were defined as those with GNI per capita between USD 1,086 and USD 4,255, while upper-middle-income economies were those with GNI per capita between USD 4,256 and USD 13,205 [16].

### Inclusion Criteria

Inclusion criteria were as follows:

1. Time period of literature search: January 1, 2010, to July 1, 2024.
2. Articles pertain to patients with DSD as defined by Hughes et al. [1].
3. Original articles and case reports describing the management and psychosocial aspects of DSD using a predefined set of keywords.
4. Articles that are scholarly, peer-reviewed publications pertaining to patients with DSD and authored by researchers from LMICs or UMICs, as defined by the World Bank (https://datahelpdesk.worldbank.org).
5. Articles in English were evaluated by two authors (S.L.S.D. and Y.G.Z.), while articles in Spanish and Portuguese were evaluated by a native-speaking co-author (M.S.).

### Exclusion Criteria

Exclusion criteria were as follows:

1. Study protocols, blog posts, and conference abstracts.
2. Studies describing syndromes such as Turner syndrome, Klinefelter syndrome, Noonan syndrome and its variants, hypogonadism (e.g., Kallmann syndrome), premature ovarian failure, polycystic ovary syndrome, ovarian and adrenal tumors, cryptorchidism, hypospadias, neonatal screening, azoospermia, male infertility, and Y chromosomal microdeletions.
3. Patients with gender identity issues in the absence of DSD.
4. Papers not available in full text.

### Selection Procedure and Quality Assessment

Duplicate records were removed using the Bramer method in EndNote [17]. Initial screening of titles and abstracts based on the established inclusion and exclusion criteria was independently conducted by two authors using Rayyan software [18]. A discussion followed the initial screening to reach a consensus on the application of the criteria. Any differences in opinions were resolved through individual discussions in each abstract. Consistency and consensus have been established in a previous literature review.

The first round of screening was conducted based on the inclusion and exclusion criteria and availability of full-text PDFs. The following data were extracted from the eligible studies: first author, title, year of publication, reference, geographical location (continent and country), article type (literature review, scoping or systematic, retrospective study of clinical management, retrospective study of molecular-genetic management, clinical and molecular-genetic case report), targeted DSD condition(s), and the number of patients studied.

A second round of in-depth screening of all full-text PDFs was conducted using a generative AI-based structured data extraction tool. Data extraction was performed using a standardized large language model-based framework designed for structured evidence capture. A predefined extraction prompt was applied uniformly to all included articles to ensure consistency across studies. Details of the prompt are provided in online supplementary Material 2. The model was instructed to extract only information explicitly reported in the source text, with inference, extrapolation, and assumption explicitly prohibited. All extracted data were consolidated into a standardized, Excel-compatible dataset for further analysis and summarized in Table 1. To assess reproducibility, the extraction procedure was repeated on a separate random subset of 10% of studies (*n* = 56) using the identical prompt and extraction schema. To evaluate the accuracy of extracted data, the random sample was independently reviewed against the source manuscripts by one of the authors (Y.v.d.Z.).

**Table 1.** Summary of in-depth screening of diagnostic modalities and effects on clinical management

​	Retrospective observational management studies		Clinical and molecular genetic management studies		Clinical and molecular genetic case reports	
	LMIC	UMIC	LMIC	UMIC	LMIC	UMIC
Total number of articles	67	54	51	99	102	162
Clinical evaluation						
Including hormonal testing, imaging, karyotyping	36	26	4	47	49	53
Including hormonal testing, imaging, karyotyping, molecular genetics	21	28	9	90	27	135
Molecular genetic tests						
Targeted single-gene testing (TGT)	18	24	44	85	45	116
Next generation sequencing (NGS)	2	3	6	13	4	22
Whole-exome sequencing (WES)	5	6	4	9	10	18
Whole genome sequencing (WGS)	0	0	1	0	0	5
Reported impact on management						
Misclassification reduction reported	7	8	7	4	16	25
Diagnostic accuracy improved by TGT	13	10	25	7	36	73
Diagnostic yield improved by NGS	3	5	6	8	13	30
Change in sex assignment	22	18	8	9	8	16
Change in hormonal management	14	14	7	6	37	79
Change in surgical timing	6	7	1	5	3	7
Reduction of diagnostic delay	6	10	15	1	10	19
Avoidance of unnecessary interventions	4	6	1	2	10	6
Multidisciplinary approach and follow-up mentioned						
MDT	19	14	12	3	13	42
Psychosocial referral	15	14	4	2	15	24
Fertility	7	4	5	6	19	23
Follow-up	14	24	3	4	27	65
Resource constraints mentioned						
Resource constraints and cost considerations	38	20	18	9	36	21

The in-depth screening resulted in pragmatic impact grading included in online supplements 4–6: grade A: quantitative evidence showing improved diagnostic yield or management change in a cohort or comparative design; grade B: consistent case-series evidence with explicit diagnostic or management impact; grade C: case-level evidence with explicit downstream clinical consequences; grade D: narrative or expert claim without supporting data; grade E: no demonstrable evidence reported. Online supplementary Table 7 provides a PRISMA ScR Extension for Scoping Reviews Checklist and Explanation.

## Results

Figure 1 illustrates the study selection process. A search conducted in July 2024 yielded 10,202 items. After the initial screening round, 735 items remained for the full-text review. Subsequently, 117 articles were excluded as they did not meet the inclusion criteria. An additional nine papers were identified through citation searching, resulting in a final total of 627 papers.

**Fig. 1.**
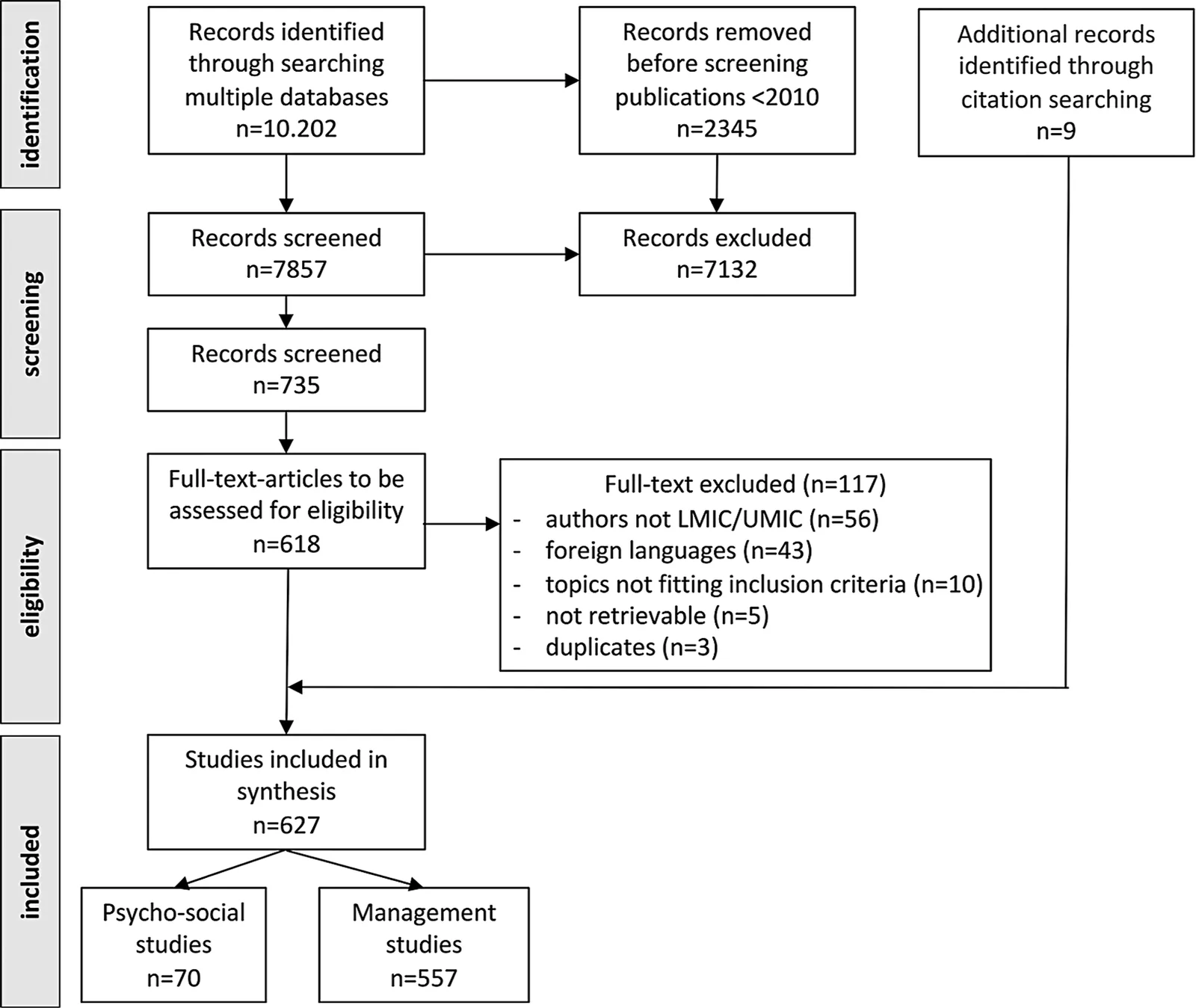
Flow diagram illustrating the study selection process for the scoping review.

Of these, 557 papers addressing various aspects of clinical and molecular genetic management of DSDs in LMICs and UMICs are included in this scoping review. The remaining 70 papers, which focused on psychosocial aspects, were analyzed in a separate study reported elsewhere [19]. The included papers were categorized and discussed under the following headings: (1) literature review (systematic and scoping), (2) retrospective clinical observational studies, (3) retrospective molecular genetic studies, and (4) clinical and molecular genetic case reports.

A heatmap (Fig. 2) summarizes the regional distribution and DSD categories. Table 2 provides a list of countries and regions affiliated with the first authors from the LMICs and UMICs.

**Fig. 2.**
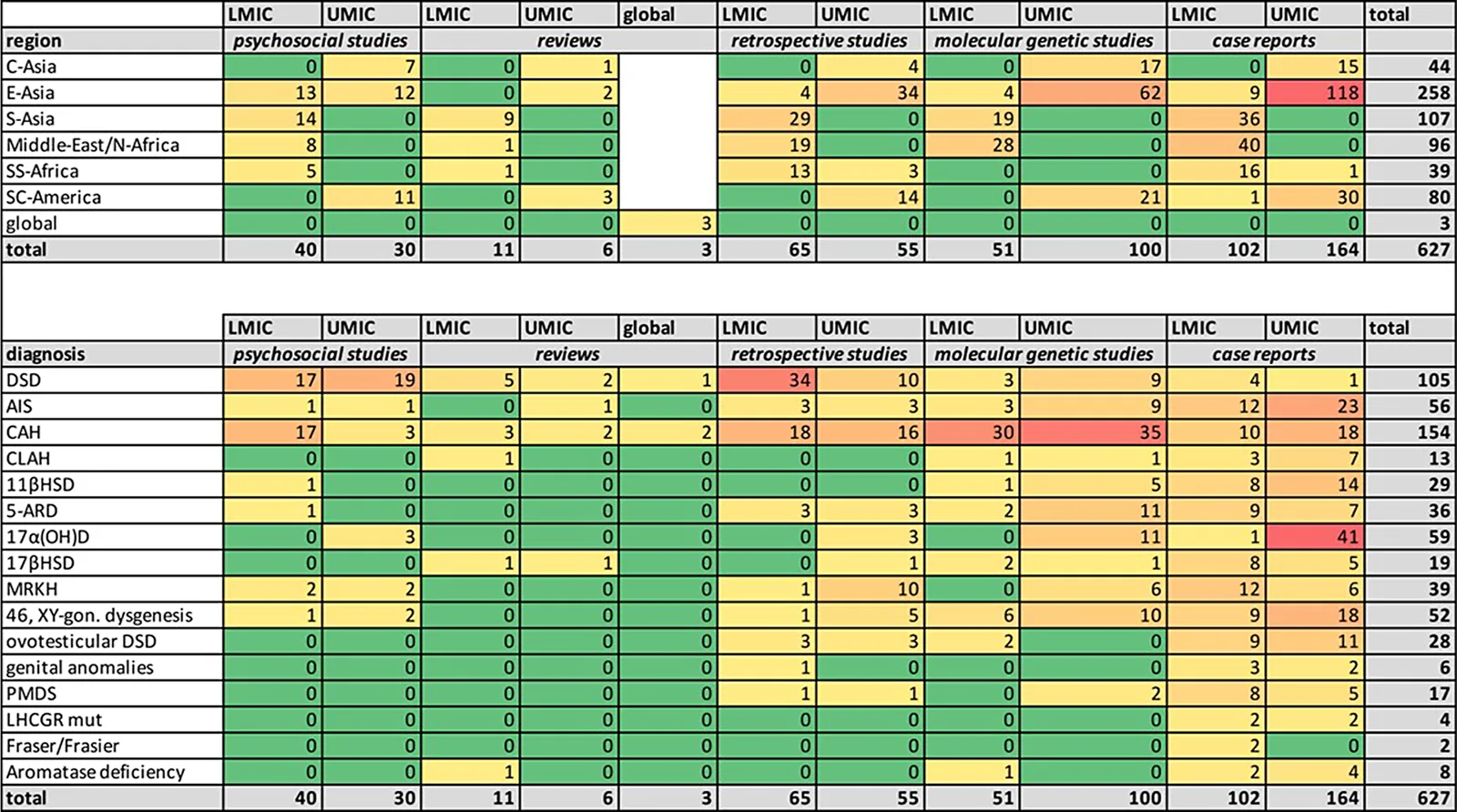
Heatmap showing the geographic distribution of paper origins and diagnostic topics addressed. DSD, disorders/differences of sex development; AIS, androgen insensitivity syndrome; CAH, congenital adrenal hyperplasia; CLAH, congenital lipoid adrenal hyperplasia; 11β-HSD, 11β-hydroxysteroid dehydrogenase; 5-ARD, 5α-reductase deficiency; 17-AHD, 17α(OH) D 17α-hydroxylase deficiency; 17β-HSD, 17β-hydroxysteroid dehydrogenase deficiency; MRKH, Mayer-Rokitansky-Küster-Hauser syndrome; 46, XY gonadal dys, 46, XY gonadal dysgenesis; PMDS, persistent müllerian duct syndrome; LHCGR, luteinizing hormone/choriogonadotropin receptor gene mutation.

**Table 2.** List of countries and regions corresponding to the first authors’ affiliations in LMIC and UMIC

Articles reviewing literature	Countries	Studies of management of DSD	Countries	Molecular genetic studies	Countries	Case reports	Countries
regions		regions		regions		regions	
**LMIC**							
S-Asia	India	E-Asia	Indonesia	E-Asia	Indonesia	E-Asia	Indonesia
​	Pakistan	S-Asia	India	​	India	​	Philippines
​	Sri Lanka	​	Pakistan	S-Asia	India	​	Vietnam
Middle-East/N-Africa	Egypt	​	Sri Lanka	​	Pakistan	S-Asia	India
SS-Africa	Nigeria	Middle-East/N-Africa	Algeria	​	Seri-Lanka	​	Nepal
​	​	​	Egypt	Middle-East/N-Africa	Algeria	​	Pakistan
​	​	​	Iran	​	Egypt	​	Sri Lanka
​	​	​	Iraq	​	Iran	Middle-East/N-Africa	Egypt
​	​	​	Morocco	​	Iraq	​	Iran
​	​	​	Syria	​	Morocco	​	Iraq
​	​	​	Tunisia	​	Tunisia	​	Morocco
​	​	SS-Africa	Cameroun	​	​	​	Syria
​	​	​	Cote d’Ivoire	​	​	​	Tunisia
​	​	​	Kenya	​	​	SS-Africa	Botswana
​	​	​	Nigeria	​	​	​	Cameroun
​	​	​	Sudan	​	​	​	Ethiopia
​	​	​	Togo	​	​	​	Sudan
​	​	​	​	​	​	​	Uganda
​	​	​	​	​	​	SC-America	Haiti
**UMIC**							
C-Asia	Turkey	C-Asia	Kazakhstan	C-Asia	Croatia	C-Asia	Bulgaria
E-Asia	China	​	Macedonia	​	Macedonia	​	Macedonia
SC-America	Brazil	​	Ukraine	​	Serbia	​	Turkey
​	Puerto-Rico	E-Asia	China	​	Turkey	E-Asia	China
​	​	​	Thailand	E-Asia	China	​	Malaysia
​	​	SS-Africa	South-Africa	​	Malaysia	​	Singapore
​	​	SC-America	Argentina	​	Thailand	​	Thailand
​	​	​	Brazil	SC-America	Argentina	SC-America	Argentina
​	​	​	Colombia	​	Brazil	​	Brazil
​	​	​	​	​	Colombia	​	Colombia
​	​	​	​	​	Costa-Rica	​	Cuba
​	​	​	​	​	Cuba	​	Dominican republic
​	​	​	​	​	Mexico	​	Ecuador
​	​	​	​	​	​	​	Mexico

Online supplementary Tables 3–6 detail the included studies by region, country, first author, title, year, journal, DSD category, number of pediatric or adult patients studied and in online supplementary Tables 4–6 pragmatic impact grading A–E is included. International collaboration was reported in 6 of the 22 literature reviews, 21 of the 121 retrospective observational studies, 26 of the 151 molecular genetic studies, and 34 of the 262 case studies.

### Literature Reviews Including Systematic and Scoping Reviews

Online supplementary Table 3 lists literature reviews and clinical guidelines relevant to the management of DSD or specific DSD conditions. Of the 20 review papers, 11 originated from LMICs and 9 from UMICs.

Systematic reviews have addressed the management and challenges of DSD in Africa [12], steroidogenic acute regulatory protein (STAR) deficiency and aromatase deficiency in India [20, 21], ethnic and national variations in congenital adrenal hyperplasia (CAH) in Brazil [22], and 17β-hydroxysteroid dehydrogenase 3 (17βHSD) deficiency in China [23]. Three multicountry reviews have provided insights into DSD in the international health literature [11] and inequities experienced by children with CAH and 11β-hydroxylase deficiency in resource-limited settings [24, 25].

Based on several papers reviewing the practical approach to the diagnosis and management of DSD in resource-limited settings, a pragmatic diagnostic algorithm suitable for low-resource settings has been developed and shown in Figure 3 and Table 3a, b [10–12, 24, 26–30].

**Fig. 3.**
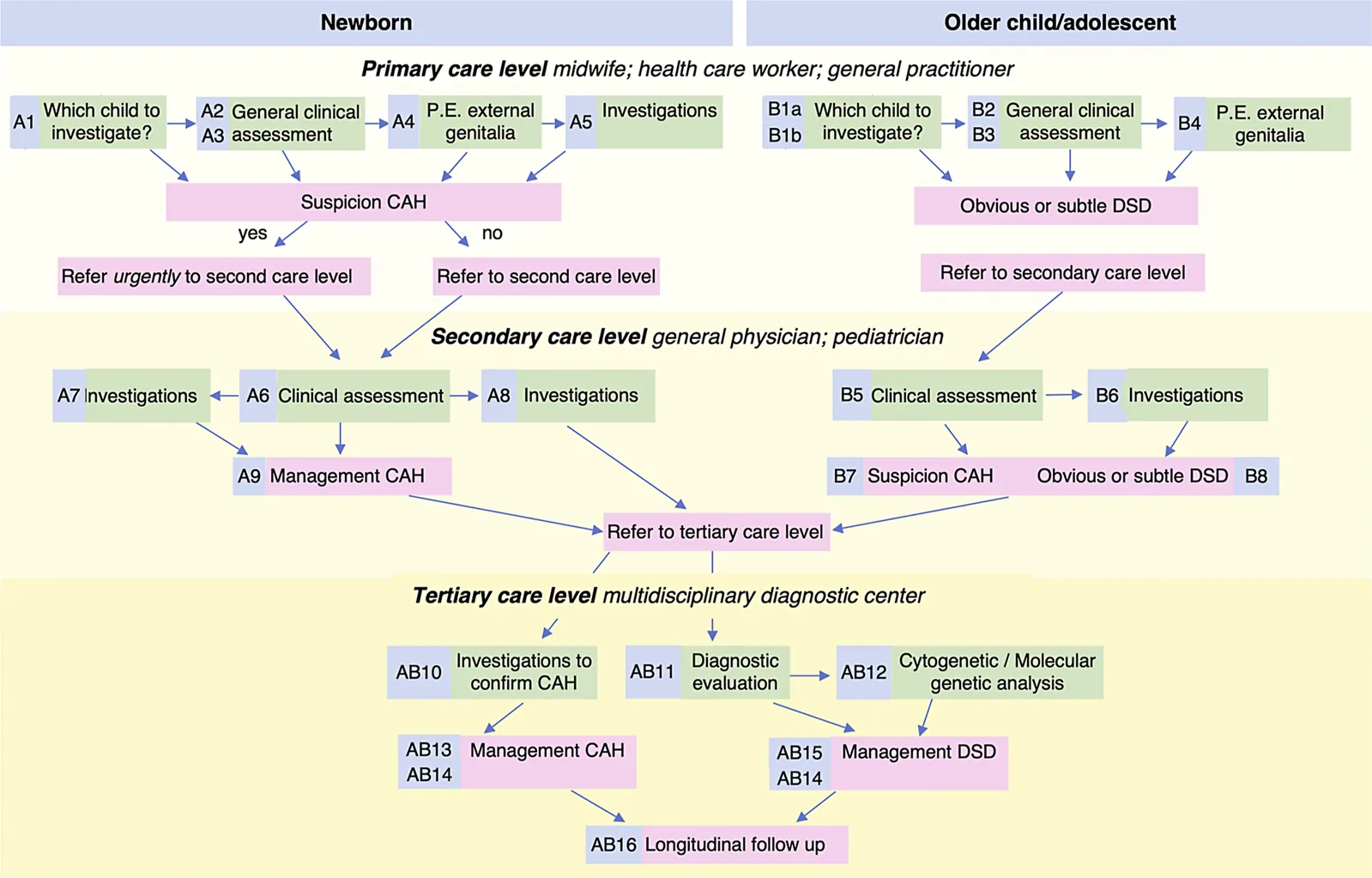
Pragmatic stepwise algorithm of diagnosis and management of newborns and older children or adolescents with DSD in a low-resource setting. The numbers refer to the explanatory Table 3a, b.

**Table 3.** Explanatory table to Figure 3

(a) Pragmatic stepwise algorithm of diagnosis and management of newborns with DSD in a low-resource setting			
A1 ***Atypical genitalia*** - Asymmetric genitalia - Bilateral undescended testes (UDT) +hypospadias grade 3 -Unilateral UDT -Hypospadias-Posterior labial fusion-Inguinal hernia in females - Isolated clitoromegaly	A2 ***Clinical assessmentHistory*** Pregnancy: - Virilizing drugs - Neonatal deaths (CAH?) - Maternal oligo/amenorrhea -Subfertility; virilization Family history: - Consanguinity - Genital ambiguity - Family members no children Perinatal history: -Apgar score; birth weight	A3 ***Clinical AssessmentPhysical examination *** - General health - Growth parameters - Shock signs - State of hydration - Blood pressure - Dysmorphic features - Small for gestational age. A4. ***Physical examination of external genitalia*** Gonads palpable uni-or bilaterally - Prader score - External Masculinization Score - Phallic length -Meatus location - Skin: texture; hyperpigmentation -Labioscrotal folds: fusion; texture	A4
A5	A6 and A7	A8	A9
**Investigations** Laboratory Serum glucose; Na; K	**Clinical assessment and investigations** Pelvic/abdominal ultrasonography Laboratory: Na ↓; K↑ 17-OH-progesterone↑	**Investigations** Pelvic/abdominal ultrasonography and Laboratory results not suggesting CAH	**Management CAH** Immediate stabilization and steroid therapy
AB10	AB11	AB12	AB12 (cont’d)
**Investigations** Pelvic/abdominal ultrasonography MRI (when indicated) Laboratory Serum electrolytes (Serum/urine adrenal steroid analysis) Karyotyping Molecular genetic analysis (when indicated^a^)	**Investigations** Pelvic/abdominal Ultrasonography MRI (when indicated) Laboratory Serum electrolytes Serum/urine gonadal/adrenal steroid analysis Karyotyping Targeted endocrine stimulation tests	**Cytogenetic investigations** (**when indicated and available**^a^) Stepwise and targeted diagnostic evaluation Chromosome testing Molecular cytogenetic: FISH/chromosomal microarray	**Molecular genetic investigations** (**when indicated and available**^a^) Quantitative fluorescent PCR Multiplex ligation-dependent probe amplification Single-gene sequence analysis NGS analysis Targeted NGS analysis panels WES WGS
AB13	AB14	AB15	AB16
**Management CAH** Continuation of steroid therapy MDC deliberation Sex assignment Uro-surgical management Family counseling	**Considerations** To defer elective surgery to adolescence with patient assent if no functional/life-threatening indication To consult (inter-) national MDC for complex cases	**Management DSD** MDC deliberation and further investigations Stepwise molecular genetic evaluation^a^ Laparoscopy/gonadal biopsy (when indicated^b^) Sex assignment Uro-surgical management Patient, family counseling	**Longitudinal follow-up** Ongoing endocrine and uro-surgical follow-up Psychological support and patient, family counseling Transition to adolescent/adult care

(b) Pragmatic stepwise algorithm of diagnosis and management of older children or adolescents with DSD in a low-resource setting			
B1a	B1b	B2	B3
**Which child to investigate** Girl Atypical genitalia Asymmetric genitalia Inguinal hernia Isolated clitoromegaly Virilization at puberty Delayed puberty Primary amenorrhea; infertility	**Which child to investigate** Boy Atypical genitalia Asymmetric genitalia Uni- bilateral undescended testes Isolated hypospadias Precocious puberty Gynecomastia Cyclic hematuria Infertility	**Clinical assessment history** Pregnancy Virilizing drugs Neonatal deaths (CAH?) Maternal oligo/amenorrhea; subfertility; virilization Family history Consanguinity Genital ambiguity Family members no children Perinatal history Apgar score; birth weight	**Clinical assessment physical examination** General health Auxology Blood pressure Dysmorphic features Midline defects
B4	B5 and B6	B7	B8
**Physical examination of external genitalia** Note: proper consent and privacy Gonads palpable uni- or bilaterally Prader score External masculinization score Phallic length Meatus location Pubic hair growth pattern Genitalia skin: texture; hyperpigmentation Labioscrotal folds: fusion; texture	**Clinical assessment and investigations** Pelvic/abdominal ultrasonography X-hand: bone age Laboratory Serum: electrolytes; 17-OH-progesterone	**Suspicion CAH** Laboratory: Na ↓; K↑ 17-OH-Progesterone↑ Stabilization and steroid therapy (when indicated)	**Investigations** Pelvic/abdominal ultrasonography Laboratory results not suggesting CAH

^a^ Characterization of cytogenetic and molecular genetic tests used in the diagnosis of DSD and details of a stepwise genetic-based diagnostic pathway in 46 XX and 46 XY DSDs are described by Özalp Kızılay et al. [31].

^b^ Indications for gonadal biopsy are described by Hannema and O’Connell [32].

### Retrospective Observational Studies on the Management of DSD Patients

A comprehensive list of 120 retrospective observational studies of DSD management is provided in online supplementary Table 4. Some studies included a brief literature review.

Sixty-seven papers from LMICs – primarily from South Asia, Middle East/North Africa, and sub-Saharan Africa – focused on single-center retrospective studies exploring DSD etiologies, clinical spectrum, and CAH management. Fifty-four papers from UMICs in East Asia and South-Central America covered a variety of DSD disorders and management practices. The detailed screening produced the following pragmatic impact grades: 13 studies grade A (LIMC 8; UMIC 5); 41 studies grade B (LMIC 23; UMIC 18); 11 studies grade C (LMIC 7; UMIC 4); 13 studies grade D (LMIC 8; UMIC 5); 44 studies grade E (LMIC 21; UMIC 23).

As exemplified in Table 1 in 36 LMIC and 26 UMIC papers, the diagnosis was based on clinical, hormonal testing, imaging studies (US/MRI), including karyotyping and rarely laparoscopy. This involved particularly steroid synthesis disorders, MRKH syndrome but also various causes of DSD, often leading to change or adaptation of hormonal management. Among papers from 21 LMIC and 28 UMIC, most focused on single-gene testing, with fewer reporting next-generation sequencing (NGS) and whole-exome sequencing (WES). These studies attributed improved diagnostic yield – both quantitatively and qualitatively – as well as greater accuracy and reduced misclassification to these methods. Additionally, a sizable portion of publications referenced modifications in hormone management, explicit alterations to sex assignment, and either adjustments to surgical timing or decisions to forego surgery altogether. Only a few reports indicated that diagnostic delays had been reduced and unnecessary interventions avoided. Ovo-testicular DSD appears more common in sub-Saharan Africa [33]. Large multicenter studies from China reported the clinical features of patients with Mayer-Rokitansky-Küster-Hauser (MRKH) syndrome [34–37], noting a lower prevalence of type II MRKH compared to European cohorts [37]. Gonadal malignancy risk was addressed in several studies [38–42]. Juniarto et al. [38] reported that, despite a lower germ cell cancer risk in Asia, DSD was a dominant risk factor. In a Chinese study, 27% of phenotypic female pediatric DSD patients with the Y chromosome showed gonadal neoplasia on histopathology [40, 41]. Not only do individuals with gonadal dysgenesis, AIS, or MRKH syndromes often experience infertility or decreased fertility, but individuals with disorders affecting steroid synthesis can also be affected. These disorders result in females in menstrual irregularities and amenorrhea and in males in small testes and hypogonadotropic hypogonadism. Nevertheless, the methods used for fertility counseling and gonadal preservation were not detailed [43–45]. Sex reassignment, primarily male-to-female, ranged from 4% to 26% across studies [46–51]. In Brazil and India, between 18% and 25% of patients with simple virilizing CAH were raised as males due to their pronounced male gender identity and pressures from their parents [52, 53]. National newborn screening programs for CAH are often lacking in developing countries, leading to delayed diagnosis and assignment of a male sex to severely virilized 46, XX infants [54, 55]. In India, patients with SRD5A2 deficiency exhibit a wide phenotypic range from females to ambiguous genitalia. Most infants diagnosed were male; individuals raised as females frequently faced gender dysphoria and transitioned after counseling [56, 57]. A major study in China found that 22 out of 103 patients were initially classified as female [58]. Multiple studies have investigated the results of genital surgery for feminization [59–63].

The surgical approaches to treating vaginal agenesis differed: in China, the Banister-McIndoe procedure involved grafts of intestinal mucosa or amniotic membrane [36, 63–65] while the Vecchietti vaginoplasty was utilized in Colombia and Brazil [66, 67]. Vaginal dilation as a nonsurgical alternative has been described in China [37] and Thailand [68]. Although numerous studies have reported large cohorts of patients with observation periods extending up to 30 years, there remains limited information regarding health outcomes, sexual function, and quality of life [69, 70]. Several publications reference the involvement of multidisciplinary teams (MDTs), fertility counseling, and psychosocial referrals (see Table 1).

A Brazilian survey found that 60% of 93 patients were abandoned, with many lacking a confirmed diagnosis [71]. Khan et al. [72] described substantial barriers to healthcare access for DSD patients in Bangladesh, including negative interactions with healthcare staff, public stigma, and institutional limitations to binary gender care, which are more pronounced in public hospitals than in private facilities.

### Retrospective Molecular Genetic Studies of DSD Patients

Online supplementary Table 5 details 150 retrospective molecular genetic studies. This includes literature reviews. Fifty-one papers from LMICs, mainly in South Asia and the Middle East/North Africa, largely focus on CYP21A2 mutations (CAH) and CYP11B1 (11β-HSD deficiency). Ninety-nine studies from UMICs (especially East Asia, South-Central America, and Central Asia) addressed CAH and DSD, as well as performed molecular analyses of androgen insensitivity syndromes and CYP17A1 variants linked to 17α-hydroxylase/17,20-lyase deficiency.

The in-depth screening resulted in a pragmatic impact grading of 11 publications: grade A (4 LMICs/7 UMICs), 47 grade B (19 LMICs/28 UMICs), 20 grade C (4 LMICs/16 UMICs), 23 grade D (14 LMICs/9 UMICs), and 49 grade E (10 LMICs/39 UMICs). According to Table 1, diagnosis was established in a limited number of studies using clinical evaluation, hormonal assays, imaging modalities such as ultrasound or MRI, and karyotyping. This involved particularly steroid synthesis disorders, often leading to change or adaptation of hormonal management. In most papers, targeted single-gene testing and less frequently NGS, WES, and WGS were reported claiming improved quantitative or qualitative diagnostic yield and accuracy and reduction of misclassification.

Numerous studies [73–77] have documented that 46, XY gonadal dysgenesis is linked to genetic factors, including genes such as DAX1, DHH, NR5A1, SOX3, SOX9, SRY, and WT1. Nagaraja et al. [78] provided a comprehensive review of monogenic causes of DSD in India with diagnostic relevance. McElreavey and Bashamboo [4] discussed in detail how consanguinity contributes to the increased prevalence of DSD in developing countries. Although the exact prevalence remains uncertain, DSD rates are higher in societies with consanguineous marriages. A recent study from Pakistan showed that 71% of children with DSD were born to mothers in consanguineous unions [79].

Several papers referenced an explicit alteration in sex assignment, as well as adjustments in the timing of surgery or decisions to forego surgical procedures. Additionally, diagnostic delays decreased and unnecessary procedures were prevented. Methods for fertility counseling and gonadal preservation were seldom detailed. However, monitoring fertility potential is crucial in patients with AR gene variants who often experience azoo-oligozoospermia, which potentially results in infertility [80].

For patients with NR5A1 mutations who may face infertility, it is advisable to offer genetic counseling and guidance about fertility options, such as preserving sperm or eggs through cryo-preservation [75, 81]. Several papers recommend raising public awareness and providing genetic counseling to help families understand the risks of consanguineous marriage and fertility concerns [82, 83]. In many papers, the importance of MDT involvement, shared decision-making, and psychosocial referral is emphasized (Table 1).

### Clinical and Molecular Genetic Case Reports

Online supplementary Table 6 summarizes 264 clinical and molecular-genetic case reports. Several of these incorporate a concise review of relevant literature. A total of 102 papers originated from LMICs, primarily from South Asia, the Middle East/North Africa, and sub-Saharan Africa, documenting novel or rare presentations and genetic findings in patients with androgen insensitivity syndrome, CAH, and MRKH syndrome. Overall, 162 case reports from UMICs, from East Asia, South-Central America, and Central Asia, frequently addressed 17α-hydroxylase/17,20-lyase deficiency, androgen insensitivity syndromes, CAH, and 46, XY gonadal dysgenesis.

The in-depth screening resulted in a pragmatic impact grading of 1 publication grade A, 60 grade B (26 LMICs/34 UMICs), 159 grade C (57 LMICs/102 UMICs), 20 grade D (9 LMICs/11 UMICs), and 24 grade E (9 LMICs/15 UMICs). As shown in Table 1 in 49 LMIC and 27 UMIC papers, the diagnosis was based on clinical, hormonal testing, imaging studies (US/MRI), and karyotyping. This involved particularly steroid synthesis disorders, AIS, MRKH syndrome, and genital anomalies, often leading to change or adaptation of hormonal management. In the remaining papers, targeted single-gene testing was most often applied, but NGS, WES, and WGS were less frequently used to describe novel gene mutations or rare pathogenic gene variants. Enhanced qualitative diagnostic results, greater accuracy, and fewer misclassifications were frequently reported. In addition, these tests contributed to explicit change of sex assignment, change in surgical timing, or avoidance of surgery. Moreover, a reduction of diagnostic delay and avoidance of unnecessary interventions was claimed albeit in small numbers. MDT involvement as well as referrals for fertility and psychosocial counseling was frequently mentioned. Information regarding sexual function and quality of life is typically not accessible for pediatric populations. Online supplement 6 indicates that numerous studies involving adults with different DSD conditions have documented the impact of diagnostic, medical, and surgical interventions on sexual function and quality of life. Of specific interest is the documentation of successful live births in women with 17α-hydroxylase deficiency through IVF, cryo-preservation, and frozen-awed embryo transfer in several papers [23, 84–86]. Similarly following bilateral gonadectomy and salpingectomy, a patient with gonadal dysgenesis was introduced to an oocyte donation program, resulting in the birth of a healthy baby [87].

## Discussion

The scope of the publications was mostly descriptive and retrospective in nature. Although the study designs were heterogeneous and lacked randomization, these studies provide insights into the clinical characteristics and diagnostic and treatment modalities of DSD, with approximately equal numbers of studies originating from the LMICs and UMICs. However, likely because of limited access to laboratory facilities and resources, the number of molecular genetic studies and case reports is significantly higher in UMICs than in LMICs.

Thorough clinical evaluation – including hormonal tests, imaging, and karyotyping – is essential to diagnose steroid synthesis disorders like 21-hydroxylase, 11β-hydroxylase, or 3β-hydroxysteroid dehydrogenase deficiencies, some of which are life-threatening. Studies show that adding molecular genetic tests, such as TGT and NGS, improves diagnostic accuracy and yield, while reducing misclassification and delays. These results were confirmed in recent studies from India indicating that the diagnostic rate of NGS is comparable to global data [88].

In addition, these tests led to changes in hormonal and surgical management and contributed to the avoidance of unnecessary interventions. The number of applications of WES and WGS was low not only because these tests are applied in new gene discovery and understanding of molecular basics but also because they are expensive [31]. Table 1 illustrates that many papers specifically discuss resource constraints and cost concerns. Economic, cultural, and sociocultural factors differ significantly across LMIC and UMIC regions. In Africa, factors such as poverty, lack of infrastructure, inadequate healthcare, reliance on folk remedies, fear of embarrassment and stigmatization, and social prejudice all contribute to delayed diagnosis and substantial challenges in managing DSD [12, 19, 46, 89].

In numerous lower- and upper-middle-income countries, neonatal screening for steroidogenesis disorders like CAH is available in only a handful of nations. In addition, there is limited availability of life-saving treatments like hydrocortisone and fludrocortisone [45, 90]. While specialized care may be available or accessible abroad, it is only an option for families who can afford it.

Genital surgery remains controversial with respect to its appropriateness, timing, and long-term outcomes [91]. This leads to clinical uncertainty in the decision-making process [92]. A recent study found that when parents and clinicians decide about genital surgery, clinicians might deliberately use uncertainty to influence parents toward what they see as the best clinical choice [93, 94]. These insights are particularly relevant in resource-limited settings, where endocrinologists and surgeons often make management decisions based on family preferences. It is well recognized that the results of feminizing and masculinizing genital surgeries may appear satisfactory at the time of the procedure; however, complications may arise a decade or more later. For example, in cases of hypospadias, issues may emerge during puberty as genital enlargement causes previous repairs to break down or later in adulthood when patients pursue sexual relationships. A recent literature review on surgical treatment outcomes for DSD reported that “normal” most often referred to phenotypic sex [95]. However, in resource-limited settings, perceptions of normality may reflect social norms, cultural beliefs, and available medical resources that differ significantly from those in affluent regions. A survey conducted in several pediatric surgical departments in India and held among stakeholders such parents, doctors, and grown-up patients with DSD revealed a predominant preference and opinion of major Indian stakeholders that support the existing approach toward DSD management, including early sex assignment and necessary medical intervention [96].

A recent expert opinion statement on genital surgery in individuals with DSD of the European Reference Network on Rare Endocrine Conditions (Endo-ERN)-ERN strongly promotes deferral of non-urgent irreversible surgical procedures until informed consent is possible [97]. Decisions about irreversible interventions should be deferred below the age of 12 years until the child is developmentally capable of expressing preferences and participating meaningfully in the decision-making process. This requires informed decision-making whereby parents must be fully informed about risks, benefits, and alternatives to surgery. Professionals within the MDT play an important role in supporting parents to make informed decisions respecting legal and ethical boundaries and the child’s right to autonomy. Exceptions to defer surgery are recognized such as medical emergencies, severe functional impairments, fertility preservation, malignancy and psychosocial considerations.

Fertility is a relevant concern for all individuals with DSD, regardless of the geographic location. However, approaches to fertility counseling and protecting gonadal function are rarely addressed.

Conversations about fertility usually focus on its impact on reproductive organs, hormone levels, and overall fertility, which can be significantly reduced by conditions like gonadal dysgenesis [98]. However, in resource-limited settings, the quality of reproductive healthcare, including fertility assessments and access to treatment, is restricted. In certain areas, laws forbid conception methods that use gamete donors or surrogates. Public awareness is encouraged, and explicit genetic counseling is highlighted to assist families in understanding the risks associated with consanguineous marriage and fertility concerns.

Current recommendations for managing DSD are primarily based on studies from wealthier countries reflecting a patient-centered approach, prioritizing the long-term well-being of individuals with DSD and human rights [97]. Patients, as well as their parents or guardians, ought to receive comprehensive support, educational resources, and detailed information regarding the diagnosis and available treatment options [99, 100]. Decisions should be made collaboratively at a multidisciplinary level with the inclusion of specialist physicians specialized in pediatric and adult care of DSD (including at least an endocrinologist, (uro-)surgeon, psychologist, and ethicist) and using a shared decision-making format with both patients and parents [97, 99–103].

Several authors have emphasized the need to establish regional centers of excellence, public awareness programs, and healthcare provider training initiatives – including standardized psychological evaluation tools – to optimize DSD management [50, 98]. Factors such as poverty, lack of infrastructure, inadequate healthcare, reliance on folk remedies, fear of embarrassment and stigmatization, and social prejudice all contribute to delayed diagnosis and as explained in more detail in the accompanying publication [19] constitute substantial challenges in managing DSD. Even in large regional centers, the etiological heterogeneity is high, and the number of patients is still small, limiting the buildup of sufficient expertise, specifically for complex cases. In addition, numerous studies have noted that molecular genetic testing can be quite expensive [31]. As shown in online supplements 4–6, 15% of all studies resulted from fruitful collaboration between LMIC/UMIC centers and institutions in more affluent regions, such as the USA, Europe and Australia.

Learned pediatric endocrinological societies, such as the African Society of Paediatric and Adolescent Endocrinology (ASPAE), the Arab Society for Paediatric Endocrinology and Diabetes (ASPED), the Asia Pacific Paediatric Endocrine Society (APPES), the Indian Society for Pediatric and Adolescent Endocrinology (ISPAE), and the Latin American Society of Pediatric Endocrinology (SLEP), are very instrumental in developing context-specific guidelines and consensus frameworks that reflect cultural realities. Moreover, international registries, including the I-DSD consortium [104], and learned societies such as the European Society for Paediatric Endocrinology (ESPE), the Pediatric Endocrine Society (PES), and the Global Pediatric Endocrinology and Diabetes (GPED), provide a crucial function in global collaborative efforts providing balanced, disease-oriented, and patient-centered expert e-learning and e-consultation programs including background knowledge and in-depth understanding of the pathophysiology and evidence-based management of DSD. This will improve the effectiveness and efficiency of care as well as community satisfaction and empowerment [105–108].

### Limitations of the Study

While we reviewed multiple databases, excluding journals from other databases may have introduced bias into our results. Given the marked etiological heterogeneity of DSD and the small number of patients per center or region, most studies are observational or descriptive in nature. The volume of publications prevented detailed reporting of individual study results. Despite these limitations, we believe that the studies included in this review provided a comprehensive overview of the current literature on DSD in LMIC and UMIC.

## Conclusions

For the last 13.5 years, most research on DSD management in low- and upper-middle-income countries has focused mainly on regions in East and South Asia, along with South and Central America. Individuals with DSD in these areas encounter a range of challenges, frequently resulting in postponed access to medical care or inconsistent adherence to treatment protocols.

We advocate the development of context-specific guidelines tailored to LMIC and UMIC, considering their sociocultural environments and resource constraints. Furthermore, promoting international collaboration in education, training, and consultation will be highly beneficial for improving the care of individuals with DSD.

## Acknowledgment

We thank Michèle Belder, medical illustrator, for design of figures and tables.

## Conflict of Interest Statement

The authors have no conflicts of interest to declare.

## Funding Sources

No sponsors or funding contributed to this study.

## Author Contributions

All authors contributed to the conceptualization and design of the study. W.B. conducted the literature search; Y.G.Z. and S.L.S.D. screened the references, extracted data, assessed the quality of the included studies, and jointly analyzed the data. S.L.S.D.: drafted the manuscript. A.U., M.S., S.M., S.H., and R.S. contributed to the writing, reviewing, and editing of the manuscript. All authors contributed to data analysis and approved the final review.
